# Projected hydrological responses to climate change in a high-mountain river basin based on RCM simulations

**DOI:** 10.1038/s41598-026-52852-6

**Published:** 2026-05-12

**Authors:** Adnan Khan, Fiza Gul, Muhammad Fahad Ullah, Hassan Ayaz, Mahmood Ahmad, Feezan Ahmad, Sabahat Hussan, Zsolt Tóth

**Affiliations:** 1 Key Laboratory of Advanced Civil Engineering Materials of Ministry of Education, School of Materials Science and Engineering, Tongji University, Shanghai, 201804 China; 2 University of Peshawar, Peshawar, Pakistan; 3 Department of Disaster Mitigation for Structures, College of Civil Engineering, Tongji University, Shanghai, 200092 China; 4 Department of Geotechnical Engineering, Tongji University, Shanghai, China; 5 Institute of Energy Infrastructure, Universiti Tenaga Nasional, Kajang, 43000 Malaysia; 6 Department of Civil Engineering, University of Engineering and Technology Peshawar (Bannu Campus), Bannu, 28100 Pakistan; 7 State Key Laboratory of Coastal and Offshore Engineering, Dalian University of Technology, Dalian, 116024 China; 8 Department of Civil Engineering, HITEC University, Taxila, 47040 Pakistan; 9 Faculty of Wood Engineering and Creative Industries, University of Sopron, Sopron, Hungary

**Keywords:** Chitral River Basin, Streamflow projection, Snowmelt hydrology, SWAT, CORDEX-South Asia, Bias correction, Quantile delta mapping, Climate sciences, Environmental sciences, Hydrology

## Abstract

This study assesses future hydrological responses of the Chitral River Basin (CRB), a high-mountain, glacier-fed catchment in northern Pakistan, under climate change. The Soil and Water Assessment Tool (SWAT) was forced with bias-corrected outputs from three CORDEX regional climate models under Representative Concentration Pathway 4.5 (RCP4.5) and Representative Concentration Pathway 8.5 (RCP8.5) scenarios for the period 2010–2099. Projected temperature increases range from 2.34 °C to 5.23 °C by the late century, while precipitation changes vary between 2.42% and 6%. These changes induce a shift in seasonal streamflow, with peak discharge advancing to June-July. Simulated streamflow responses indicate that warming may alter seasonal runoff timing through enhanced snow and ice melt processes. However, because the adopted SWAT configuration assumes static glacier area, projected late-century reductions should be interpreted as climate-driven hydrological responses rather than direct simulations of progressive glacier depletion. The results highlight substantial uncertainty in future annual flow magnitude across climate models and bias-correction methods, while consistently indicating sensitivity of runoff timing to climatic warming. These findings underline the need for adaptive water-management strategies in the Chitral River Basin.

## Introduction

 Water is indispensable for sustaining all life forms and plays a pivotal role in industrial development, agriculture, potable water supply, and recreational activities^1^. However, only about 2.5% of the total global water is freshwater, and nearly 70% of that is sequestered in glaciers and polar ice sheets. This severely limits the amount of usable water, making its availability a critical issue, particularly under the looming threat of climate change^2,3^. Earth’s climate has historically alternated between glacial and interglacial states. In recent decades, however, the rate of warming has accelerated markedly. This has been characterized by rising atmospheric and ocean temperatures, increased melting of snow and ice, and a corresponding rise in sea levels^4–7^. As stated in the IPCC Sixth Assessment Report (AR6), global average surface temperatures are projected to rise by 1.5 °C between 2030 and 2035. A significant increase from the 0.75 °C cited in the Fourth Assessment Report (AR4), thereby amplifying concerns around climate intensification^8^. In South Asia, average temperatures in coastal regions have risen by 0.6–1.0 °C since the early 1900s, and in Pakistan, a rise of 0.6 °C was recorded between 1901 and 2000^9^.

The increasing global population adds further stress to freshwater resources. Over the last 50 years, the world population has expanded from 3 billion to 6.5 billion, and projections suggest it could reach 8 billion by 2025 and 9 billion by 2050. Currently, irrigation alone accounts for roughly 70% of all freshwater withdrawals worldwide^10^. Pakistan, like many South Asian countries, is heavily dependent on temperature-dependent glacier–snowmelt-dominated river systems. Cryospheric processes dominate the Upper Indus Basin (UIB) and supplies vital water through the Indus River and its network of tributaries^11^.

Numerous investigations have evaluated the hydrological consequences of climate change using Regional Climate Models (RCMs). For instance, Andrea et al. (2014) utilized projections from the IPCC’s Fifth Assessment Report across three Representative Concentration Pathways (RCP2.6, RCP4.5, and RCP8.5). They forecasted an advancement in snowmelt timing, intensified glacial melt, and a gradual decline in streamflow owing to thinning ice layers^12^. Similarly, Gerhard et al. (2014) predicted temperature rises of approximately 2.2 K during 2031–2060 and up to 3.5 K by 2070–2099. This warming is accompanied by a decrease in precipitation ranging from 12% to 35%, ultimately resulting in marked reductions in streamflow within the Upper Jordan River Basin^13^. In another study, Aijing Zhang et al. (2016) implemented the SWAT model to simulate hydrological behavior in the Heihe River Basin (HRB). They projected an increase in summer precipitation under future climate scenarios, with potential implications for regional irrigation practices^14^. Similar multi-RCM climate impact assessments in transboundary river basins have been reported by Skoulikaris et al., highlighting the role of ensemble climate projections in hydrological uncertainty analysis^15,16^.

For Pakistan’s Upper Indus Basin (UIB), numerous studies have documented notable alterations in seasonal streamflow patterns, increased flood frequency, and reduced water availability. According to Zu et al. (2024), peak flows are shifting earlier in the season (from May–June to July–August), with their magnitude increasing by 50–100%^17^. Remote climate drivers, such as tropical–Arctic teleconnections and cyclone-induced ocean dynamics, influence regional precipitation and cryospheric processes in high-latitude and mountain systems^18,19^. Long-term river basin evolution, as evidenced by sediment provenance in the Yellow River, further confirms that hydro-climatic shifts fundamentally reshape drainage networks and sediment routing^20^. Shabeh et al. (2019) examined the influence of glacial loss on the Jhelum, Kabul, and Indus rivers, concluding that a 25% reduction in glacier volume is plausible under a 1.5 °C warming scenario^21^. Farooq et al. (2016) used GCMs and RCMs for the Naran Basin to project changes in precipitation and sediment yield under warming trends^22^. Similarly, Shaukat Ali et al. (2015) indicated that under RCP4.5 and RCP8.5, streamflow could rise by 12–20% by 2100, although both pathways may overpredict precipitation and temperature in the UIB region^23,24^.

Climate variability is already influencing water resources across Pakistan. The national average temperature has climbed by approximately 0.5 °C per decade over the last 50 years^25^. Glacial retreat, particularly in Khyber Pakhtunkhwa (KPK), elevates the likelihood of flood hazards, while both surface and subsurface water reserves are rapidly depleting. In 2016, per capita freshwater availability fell below 1,000 cubic meters and has continued to decline^25^. Given these threats, evaluating future water availability with advanced modeling tools is essential for devising sustainable water management strategies. Projected hydrological responses in high-mountain river basins reflect the combined effects of altered snowmelt and ice-water processes, eco hydrological feedbacks, and intensifying climate extremes. This is supported byevidence from fluid-ice interactions, polar climate dynamics, and constrained Earth system modeling^26–29^. Advances in remote sensing, deep learning, and high-resolution atmospheric and surface observations improve the representation of soil moisture, convection, and energy exchanges in RCM-based hydrological simulations^30–34^. Isotope-based studies further constrain precipitation sources, groundwater-surface water interactions, and water cycling in mountainous and arid basins, enhancing the physical realism of future hydrological projections^35–39^. Climate-driven vegetation change, earlier leaf-out, and large-scale system feedbacks regulate basin-scale water balance and runoff responses under warming conditions^40–43^. Furthermore, game-theoretic bargaining approaches offer innovative solutions for transboundary water allocation under risk and uncertainty^44–46^. Finally, floods, landslides, debris flows, subsidence, and dike instability can amplify hydrological risks in high-relief terrain, highlighting the need for integrated RCM-based assessments^34–46^. These dynamics are further substantiated by recent empirical evidence^47–49^.

The Chitral River Basin is a high-mountain tributary of the Kabul River system in which runoff is strongly controlled by snowmelt, glacier melt, and temperature seasonality. Previous hydro-climatological work in the basin reported a mean annual flow of about 288 ± 25 m³ s⁻¹ at the Chitral gauging station, a runoff depth of about 735 mm, and annual precipitation totals of approximately 464 mm at Chitral and 580 mm at Drosh, while also emphasizing that low-elevation valley stations underestimate high-altitude precipitation inputs. These characteristics make the basin highly sensitive to warming-induced shifts in runoff timing and melt-season hydrology. Although climate-change impacts have been widely studied in the wider Upper Indus Basin, basin-specific assessments for the Chitral River Basin remain limited, especially studies combining multiple CORDEX regional climate models, explicit bias correction, and distributed hydrological modeling. The present study addresses that gap by applying SWAT to the Chitral River Basin using three CORDEX-South Asia regional climate models under RCP4.5 and RCP8.5 to evaluate future streamflow timing and annual water availability under a static-glacier-area assumption.

## Study area and data collection

### Study area

**Fig. 1:**
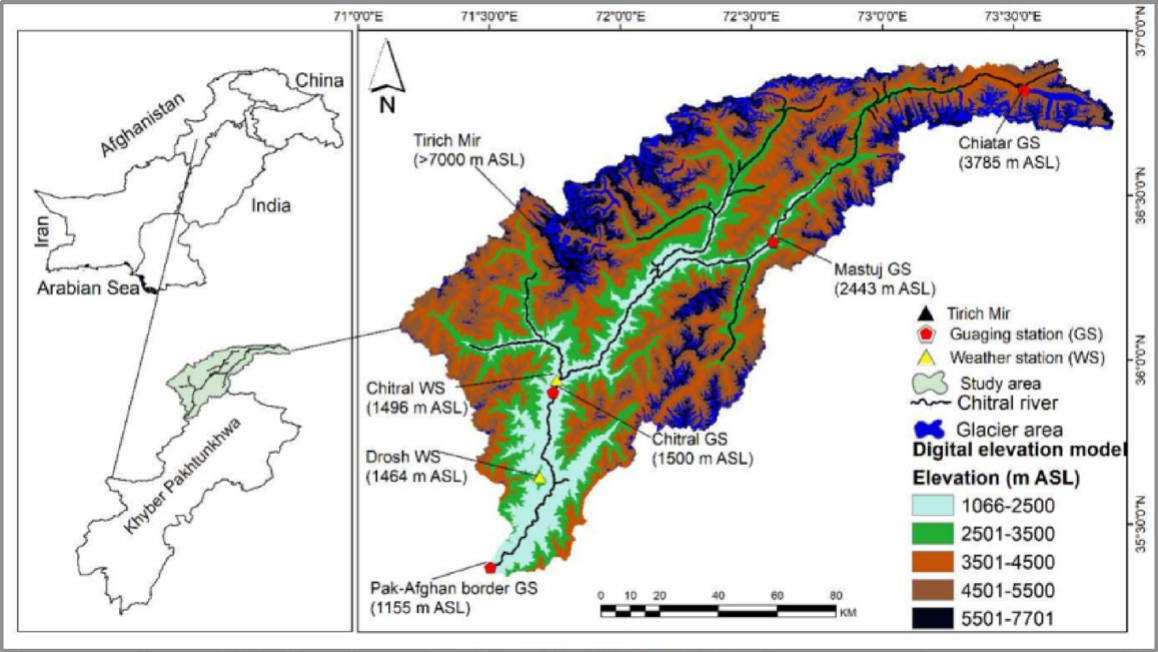
Location map and digital elevation model of the study area^24^.

The Chitral River Basin, situated in the Hindukush mountains of northeastern Pakistan, was chosen as the focus of this study. The Chitral River is a significant indirect tributary of the Indus River, originating near the Baroghil Pass in the Hindukush Mountains. It flows from the Kunhar River, which is itself a tributary of the Kabul River in Afghanistan. A location map of the Chitral River Basin, along with the Digital Elevation Model (DEM), zonal classification, and key topographic features, is shown in Fig. 1. The uppermost part of the Chitral River, known as the Yarkhun River, is located between the towns of Mastuj and Chitral, and further downstream, it is referred to as the Chitral River. The river lies within the geographical coordinates of 35˚50’ N and 71˚48’ E^24^. Five meteorological stations situated within or adjacent to the basin (Fig. 1) provided the observational data. However, we use two meteorological stations, Chitral meteorological stations and Drosh meteorological stations in this study because of consistent data availability. The river is gauged at a flow gauge (FG) located in the town of Chitral (Fig. 1) on the river bridge.

Over the historical period, the Chitral River Basin exhibits a strong mismatch between outlet precipitation and basin runoff because most available weather stations are located at low valley elevations, whereas a large part of the basin lies at much higher altitudes where snow accumulation is substantial. Previous basin-scale analysis reported a mean annual flow of approximately 288 ± 25 m³ s⁻¹ at the Chitral gauging station, equivalent to an annual runoff depth of about 735 mm. Annual precipitation totals are about 464 mm at Chitral and 580 mm at Drosh, but these station values do not fully represent the high-altitude precipitation contributing to basin runoff. This supports the interpretation that streamflow in the basin is strongly sustained by snow and glacier melt from higher elevations; the remaining water balance component is attributed to basin wide evapotranspiration losses. On July 8, 2019, a glacial lake outburst flood in the Golen Gol region of Lower Chitral caused widespread devastation. The flood, triggered by the sudden bursting of the Jam Ashpar glacier, destroyed five bridges, toppled power poles, and submerged roads and farmlands. According to the Khyber Pakhtunkhwa Provincial Disaster Management Authority (PDMA), the event caused significant damage to local infrastructure and livelihoods (Dawn, 2019).

The Chitral River is part of the transboundary Chitral-Kunar-Kabul River system. However, during the calibration and validation baseline considered in this study, no major regulating reservoir was operating upstream of the Chitral gauging station; therefore, the observed discharge is treated as predominantly natural flow.

### Datasets

#### Hydro-meteorological data

The uppermost section of the Chitral River is known as the Yarkhum River, between the towns of Mastuj and Chitral, and downstream it is called the Chitral River, located between 35˚50 and 71˚48. A distributed network of meteorological stations spans the northern region, aiding basin-wide identification and analysis. It is maintained and recorded by two public sector entities, namely the Pakistan Meteorological Department (PMD) and the Water and Power Development Authority (WAPDA). The meteorological observations from these stations include daily precipitation, maximum, and minimum temperature values for 30 years, to substantiate the impacts of climate change in this study region.

The Pakistan Meteorological Department (PMD) provided daily precipitation, Tmax, and Tmin data for two stations (Chitral, Drosh) for 1976–2005. Daily discharge data for the Chitral gauging station were obtained from the Water and Power Development Authority (WAPDA) for 1977–2004. Although WAPDA’s systematic meteorological records began in the mid‑1990s, their streamflow gauging records extend back to the 1970s. Therefore, only WAPDA’s meteorological data were unavailable; their discharge data were used for calibration and validation. An inventory of all PMD stations used in this study to date is given in Table 1.

**Table 1:** List of Meteorological Observatories in the Chitral River Basin.

Sr.#	Station name	Agency	Lattitude	Longitude	Altitude (m.asl)
1	CHITRAL	PMD	35.85	71.83	1496
2	DROSH	PMD	35.57	71.78	1464

#### Climate model data

The Coordinated Regional Climate Downscaling Experiment (CORDEX), initiated by the World Climate Research Program (WCRP), aims to develop a global framework of regional climate projections based on the Fifth Assessment Report of the Intergovernmental Panel on Climate Change (IPCC). The project produced global climate model predictions at regional scales for impact analysis. It made a pool/ensemble of climate model dynamically and statistically downscaled GCM run from the CMIP5 database with an initial resolution of 0.44–50 km.

For this study, the selected Regional Climate Models (RCMs) and their GCM forcing data were obtained from the Earth System Grid Federation (ESGF), with a focus on the South Asian domain. These datasets, formatted in netCDF, provide projections under different Representative Concentration Pathways (RCPs). Specifically, RCP 4.5 (4.5 W/m²) and RCP 8.5 (8.5 W/m²) (Table 2) scenarios were used, as they represent mid-range and high-end emission trajectories, respectively, and are considered the most plausible for long-term forecasting by 2100.

**Table 2:** RCMs model dataset.

Institute	RCM	Driving GCM	Emission scenario
CSIRO	CCAM	MPI-ESM-LR	RCP 4.5 & 8.5
SMHI	RCA4	EC-EARTH	Same as above
MPI-CSC	REMO2009	MPI-M-MPI-ESM-LR	Same as above

For the present study, RCM datasets enable the identification of climatologically significant changes at fine resolutions on a global scale. GCMs provide boundary conditions; RCMs dynamically downscale these to finer resolutions for impact‑oriented studies .

This research considers different RCPs to capture the range of plausible future climate outcomes. Given the similarities between RCP4.5 and observed CO₂ emission trajectories between 2005 and 2012, RCP4.5 was prioritized alongside RCP8.5, which represents more extreme warming scenarios. RCP2.6 was excluded due to its reliance on sustained and unrealistic global CO₂ reductions. Both RCP4.5 and RCP6.0 depict moderate climate futures, but RCP4.5 was selected because it aligns with observed emission rate trends (1.5% annually)^50^.

#### Land use/land cover data

**Fig. 2:**
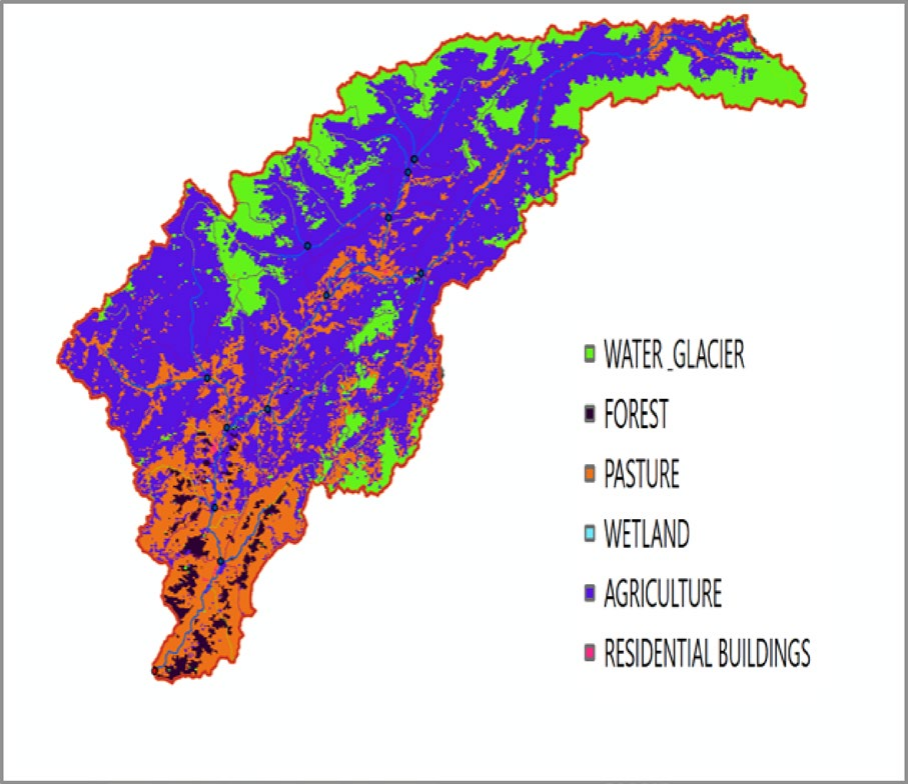
Land cover classes observed in CRB.

LULC is the primary input data for SWAT modelling, as variations in LULC can substantially affect runoff, evapotranspiration, and other hydrological parameters^51^. LULC data was obtained from the USGS Global Land Cover System, later reclassified to suit SWAT model categories at 1 km spatial resolution, and further clipped to the shape file to get a map of the desired land use for CRB. Based on land cover and Land use, the study area was divided into six major categories, which were then reclassified according to their hydrologic properties and SWAT requirements. For each land category, the model provides a unique four-letter code. The division of these classes is shown in Table 3; Fig. 2, where major classes were forest mixed, forest deciduous, range grasses, agricultural land, row crops, etc. As we can see in Table 3, Glacier and permanent snow cover account for approximately 18% of the basin area. Vegetation-covered land (forest, pasture, and agriculture) constitutes about 82%, reflecting strong cryosphere control on basin hydrology.

**Table 3:** Land use classification of Chitral River Basin with percentage area covered.

S. #	LULC Classes	SWAT classes	% Area covered
1	Water and glacier	WATR	18.03
2	Forest	FRST	2.34
3	Pasture	PAST	20.25
4	Wetlands	WETL	0.022
5	Agricultural land	AGRL	59.24
6	Residential building	URHD	0.088

#### Soil classification

**Fig. 3:**
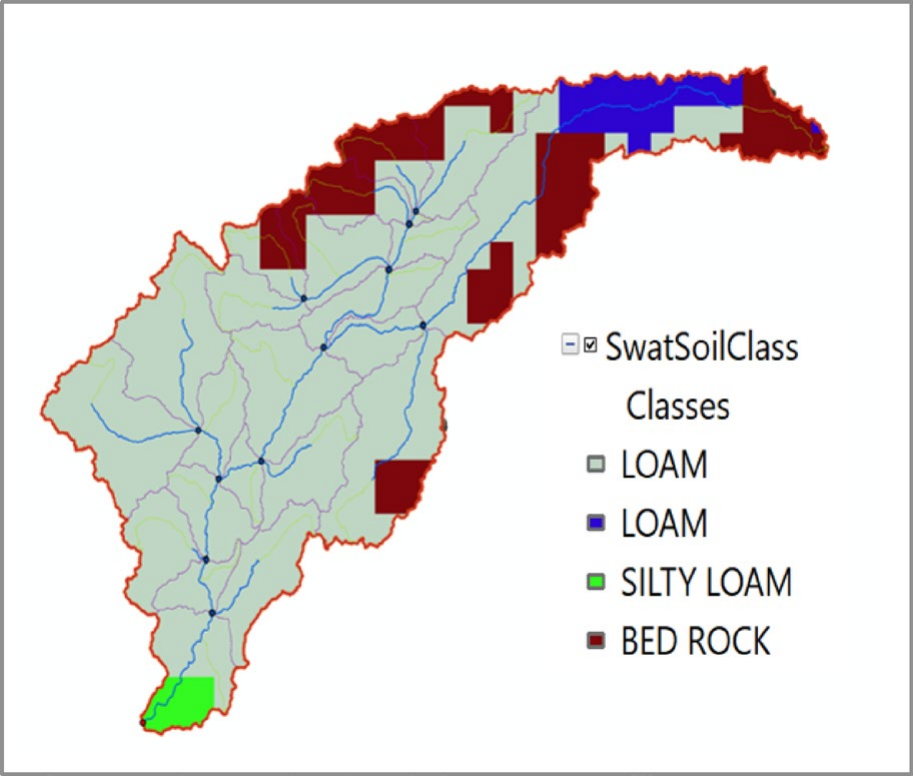
FAO Soil classification CRB.

The hydrological performance of soil is well described by its physical properties. Improved hydrological model parameterization, such as the stepwise grid-Xin’anjiang approach, better represents spatial heterogeneity in runoff processes^52^. Meanwhile, quantitative assessments of coastal land-use changes and synoptic drivers of extreme precipitation reveal how anthropogenic and climatic factors jointly influence regional water hazards^53,54^.Different soil physicochemical and textural properties are required for the SWAT model. These models include available water content, soil texture, bulk density, hydraulic conductivity, and carbon content for each layer of different soil types. For this study, FAO/UNESCO soil data were used, projected to UTM and 90 m*90m resolution, and then applied in a model for Hydrological Response Unit (HRU) analysis. After importing the soil map into the Arc-SWAT interface, four soil types were delineated in the basin, as summarized in Table 4. Similarly, Soil characteristics are also mentioned in Fig. 3.

**Table 4:** Soil texture classification of Chitral River Basin with percentage of particles.

S. #	FAO soil type	% area covered	Texture	Clay%	Silt%	Sand%
1	I-B-U-2c-3503	77.18	LOAM	26	30	44
2	I-X-2c-3731	4.91	LOAM	22	33	45
3	Xh18-bc-3870	1.69	SILT LOAM	21	54	26
4	GLACIER-6998	6.18	UWB	5	25	70

## Methodology

### SWAT model setup

The SWAT model operates as a distributed, physically based tool developed to simulate long-term hydrological dynamics and sediment transport in watershed systems. Its primary goal is to assess the influence of land use, management practices, and climatic variables on processes such as water quantity, crop productivity, nutrient fluxes, and sediment yield. Snow and glacier melt are simulated using SWAT’s temperature-index (degree-day) approach, where melt rates are governed by calibrated temperature thresholds. Glacierized areas are represented as snow- and ice-dominated HRUs, allowing meltwater contribution to streamflow to be indirectly captured. The SWAT model used in this study does not explicitly simulate dynamic glacier mass balance or glacier retreat. Glacier melt is simulated using a temperature-index (degree-day) approach, while glacier area is kept constant throughout the simulation period. Land use and glacier extent were assumed to remain static; therefore, potential future glacier retreat, glacier mass depletion, and vegetation shifts were not dynamically simulated. Accordingly, projected streamflow changes should be interpreted as climate-driven hydrological responses under a static-glacier-area assumption, rather than as explicit simulations of future glacier retreat or glacier mass loss. By partitioning watersheds into sub-units, the model enhances simulation detail and computational efficiency. It supports future scenario analysis using diverse datasets, including land use, topography, climate, and soil data. SWAT modules address hydrological cycles, crop growth, water quality, and nutrient movement, all of which are validated in numerous case studies^55–58^. Reservoir operation and water abstraction modules were not activated in SWAT due to the lack of reliable operational data for existing or planned infrastructure. Consequently, the simulations represent climate driven hydrological responses under naturalized flow conditions.

Model implementation details: SWAT v.2012 (ArcSWAT 2012.10_2.16) was used. DEM source: SRTM (30 m resolution). HRU thresholds: land use 5%, soil 10%, slope 10%. Elevation bands: 5 bands (1000–1500, 1500–2500, 2500–3500, 3500–4500, > 4500 m). Snow parameters: temperature‑index method with SMFMX = 4.5 mm/°C/day, SMFMN = 2.0 mm/°C/day, Tmelt = 0.5 °C, and a snowpack temperature lag factor of 0.5.

### Watershed delineation

**Fig. 4:**
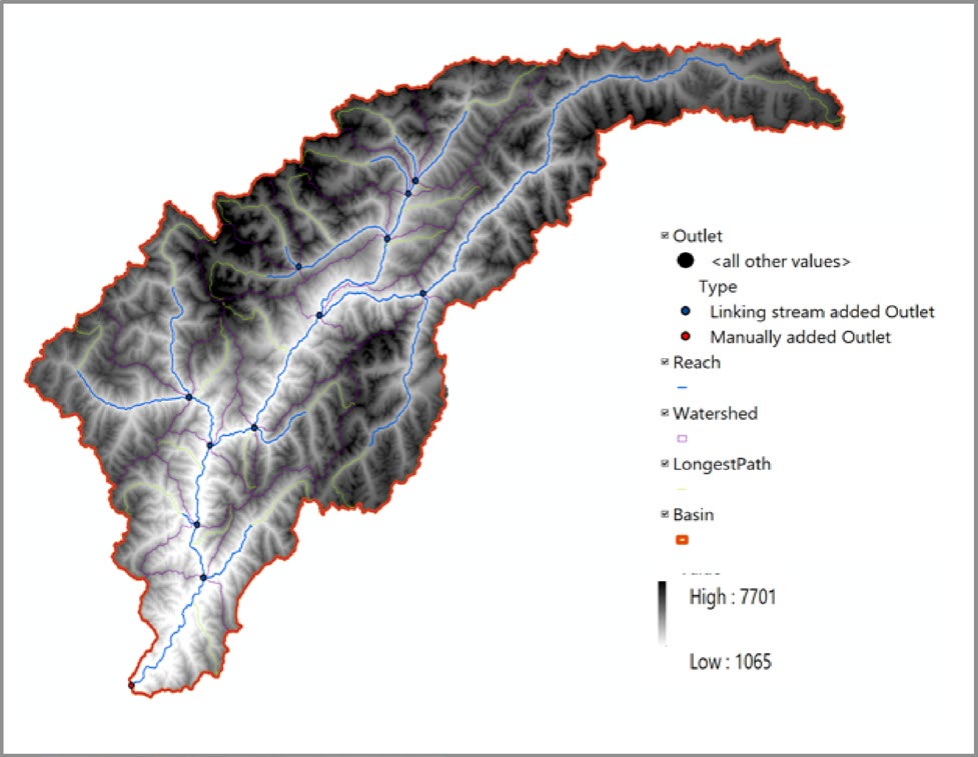
Watershed delineation of CRB.

Establishing watershed boundaries is a foundational step in SWAT-based hydrological modeling. In this study, the digital elevation model (DEM) was analyzed to extract topographic attributes, such as slope direction and accumulation, which are essential for defining stream networks and watershed edges. The DEM was projected using the UTM coordinate system (Zone N43), consistent with local geospatial standards in Pakistan. The catchment was segmented into 23 sub-basins based on elevation and hydrological flow paths (Fig. 4). Parameters such as stream delineation, outlet positioning, and inlet identification were systematically defined to produce accurate Hydrological Response Units (HRUs).

### Slope classification and HRU analysis

**Fig. 5:**
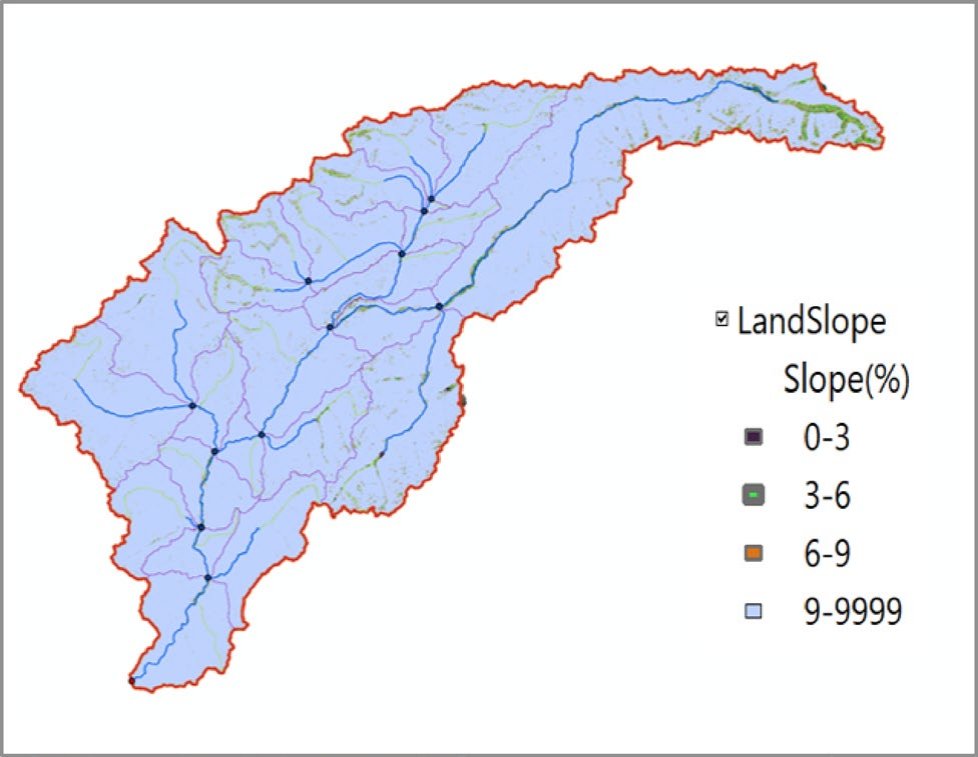
Land slope classification of CRB.

SWAT organizes spatial data into Hydrological Response Units (HRUs), which integrate slope, soil, and land use information within each sub-basin. Following watershed delineation, slope classes were derived from DEM elevations (Fig. 5), while land use and soil types were classified based on standardized SWAT input formats (Table 5). These datasets were aligned under the UTM N43 coordinate system. HRUs were then created using the multi-slope method, allowing detailed spatial variability within sub-basins. SWAT can define HRUs either by unique land/soil combinations within sub-basins or by threshold-based filtering to reduce computational load.

**Table 5:** Soil texture classification of Chitral River Basin with percentage of particles.

S. No	Slope class	Upper-lower limit	% Age area coverage
1	Class 1	0–3	0.59
2	Class 2	3–6	1.36
3	Class 3	6–9	1.7
4	Class 4	9-9999	96.35

### Climate data and quality control

The statistically downscaled climate data were categorized into three 30-year intervals: 2010–2039, 2040–2069, and 2070–2099. After which, leap years were excluded to standardize the dataset for trend assessment and bias adjustment. The study incorporated outputs from three regional climate models (RCMs), each covering the periods above. These RCMs provided daily records of minimum and maximum temperatures as well as precipitation data relevant to selected meteorological stations.

Compared to general circulation models (GCMs), these RCMs offer a finer spatial resolution (0.44° × 0.44°), enabling more localized climate projections. The model outputs are aligned with two Representative Concentration Pathways (RCPs): RCP4.5, reflecting moderate emissions, and RCP8.5, representing high emissions scenarios. These pathways are widely used to evaluate future climate trends because they incorporate a range of radiative forcing levels by 2100. A comprehensive list of RCMs and their associated GCMs is provided in Table 2.

### Bias correction using quantile delta mapping (QDM) and CMhyd software

Bias correction was performed using Quantile Delta Mapping (QDM) to correct systematic distributional biases in RCM outputs. The Best Easy Systematic (BES) estimator is used for both temperature and precipitation. Although the BES method performs well for temperature, it performs poorly for rainfall^16^.

The QDM approach maintains the quantiles of projected changes while simultaneously removing systematic errors in the quantiles of the projected series relative to the observed datasets. QDM bias correction in Table 6 applied to the two IPCC emission scenarios of their respective downscaled RCM datasets for the selected time slices, i.e., 2010–2039, 2040–2069, and 2070–2099. QDM was calibrated using observed data (1976‑2005) and applied to RCM outputs for 2010‑2099. Corrections were applied monthly. Validation used a split‑sample approach: 1977‑1996 for calibration, 1997‑2004 for validation, achieving NSE > 0.85 for all variables.

**Table 6:** Bias-correction for different time slices along variables using QDM & CMhyd.

RCM	IPCC emissions scenario	Time slice	Variable	Bias-correction
CCAM	4.5 & 8.5	2010–2039 2040–2069 2070–2099	Prcp, Tmax & Tmin	Complete
RCA4	4.5 & 8.5	2010–2039 2040–2069 2070–2099	Prcp, Tmax & Tmin	Complete
REMO	4.5 & 8.5	2010–2039 2040–2069 2070–2099	Prcp, Tmax & Tmin	Complete

CMhyd was developed to simulate climate data that can effectively represent the locations of gauges used in a watershed model setup. Accordingly, climate model data were extracted and bias-corrected for each gauge location. CMhyd reads observed data in ASCII format. Each gauge’s data is saved in an individual file, organized by a location file. These location files specify precipitation and temperature data separately. The location file contains the relevant data file names and the gauges’ coordinates (LAT and LON). In the data files, the first line indicates the starting date of the time series, with subsequent lines representing daily records. Missing data (gaps) are represented by a no-data value (– 99.9 or − 99.0). The precipitation file provides one daily record (total daily precipitation in mm), and the temperature file contains daily maximum and minimum temperatures (°C). This method identifies differences between current and projected GCM simulations and applies those changes to observed time series. The correction adjusts observations according to the GCM or RCM response to climate change. For instance, if a GCM anticipates that temperatures will be 3 °C warmer in the future, then 3 °C is added to historical values to generate a new future climate series. For rainfall, a percentage change is usually applied; for example, if a 20% increase is projected, the past values are multiplied by 1.2. These delta adjustments may vary seasonally or monthly.

### Trend analysis

To assess long-term trends in hydro-meteorological parameters, the study applied the non-parametric Mann–Kendall (MK) trend test with a 5% significance threshold. The test was used to determine monotonic trends in variables such as river discharge, air temperature, and rainfall. Sen’s Slope estimator (Sen, 1968) was utilized to quantify the magnitude of change over time, while Kendall’s tau (τ) coefficient supported significance evaluation.

A linear regression model was also employed to visualize trends. Both seasonal and annual scales were examined to identify temporal patterns. The MK test, in combination with Sen’s Slope, is widely used in climate-related research to detect non-linear but consistent shifts in environmental variables^59–61^.

### Sensitivity analysis and model calibration

Sensitivity analysis plays a crucial role in identifying the most influential model parameters to adjust, given the study area’s specific characteristics. Given the inherent uncertainties in model input parameters, a systematic variation of these parameters was performed to enhance model accuracy and efficiency. This process ensures a better understanding of the system’s hydrological behavior and the model’s overall performance.

In this study, a local sensitivity analysis (one-at-a-time method) was implemented to determine the key parameters affecting streamflow simulation. These parameters were selected based on their impact on model output. To refine model accuracy, calibration and validation procedures were conducted to optimize the agreement between observed and simulated hydrological variables. Using daily discharge data from the Chitral River Station, the model was calibrated for the period 1977–1996 and validated using discharge records from 1997 to 2004.

Daily discharge data were used for calibration, with the observed mean annual flow of 274.3 m³/s serving as a reference for water balance, while daily peaks (8000–10,000 m³/s) were used to calibrate snowmelt and runoff parameters. The apparent difference between the mean annual flow and daily peak flows reflects the strongly seasonal hydrograph of this glacier‑fed basin, where > 70% of annual runoff occurs during June-August. The model was calibrated to reproduce both seasonal dynamics and peak magnitudes, not merely annual means.

For calibration, the Sequential Uncertainty Fitting (SUFI-2) algorithm within SWAT-CUP v. 5.2.1.1 was applied^62^. The parameter ranges were determined based on prior hydrological knowledge, sensitivity analysis results, and literature. Model performance was quantified using two key uncertainty metrics: P-factor and R-factor, where the P-factor represents the proportion of simulated estimates falling within the 95% Prediction Uncertainty (95PPU) band, and the R-factor reflects the discrepancy between observed and simulated values.

A local sensitivity analysis employing the One-at-a-Time (OAT) technique guided the preliminary parameter selection, which was later refined using the SUFI-2 algorithm within SWAT-CUP. To ensure that the most important factors governing runoff, infiltration, base flow, and snowmelt dynamics were adjusted to reflect the glacier-fed, high-altitude hydrology of the Chitral River Basin, the calibration was carried out using historical discharge data.

### Justification for model and scenario selection

The Soil and Water Assessment Tool (SWAT) was chosen in this research for its capacity to model hydrological processes in large, heterogeneous, and data-sparse basins over long temporal scales. SWAT provides a physically based, semi-distributed framework that incorporates glacier-fed runoff, snowmelt, evapotranspiration, soil moisture dynamics, and land-use transformation impacts, all of which are crucial in the high-altitude context of the Chitral River Basin (CRB) in contrast to other models like HEC-HMS, HBV, or VIC, which either lack representations of the snowmelt process or have limited parameter flexibility. It is well-suited to the varied topography and land-cover variations observed in the CRB, owing to its integrated GIS-based HRU delineation and demonstrated performance in earlier Himalayan and Upper Indus Basin studies.

This study employs CORDEX-South Asia regional climate model outputs driven by CMIP5 RCP4.5 and RCP8.5 scenarios. Although SSP-based CMIP6 projections are increasingly adopted, high-resolution SSP-driven CORDEX simulations with consistent multi-model availability remain limited for the study region. The selected RCPs represent intermediate and high radiative forcing pathways and remain widely applied in regional hydrological impact assessments. For climate forcing, three Regional Climate Models (CCAM, RCA4, REMO2009) were chosen due to their availability under the CORDEX-South Asia domain, their proven performance in similar glacio-hydrological regions, and their diversity in representing atmospheric processes. Using multiple RCMs ensures a broader capture of uncertainty, which is essential for robust hydrological forecasting. To correct inherent biases in raw RCM outputs, Quantile Delta Mapping (QDM) was applied as an advanced bias correction method that preserves the statistical distribution of extremes, which are particularly important in flood-prone mountainous regions. In contrast to simpler methods such as Delta change or linear scaling, QDM preserves the integrity of peak-flow events, aligning with the study’s objective of assessing seasonal shifts and hydrological extremes under future climate scenarios.

This combined model setup, SWAT with multi-RCM input and QDM bias correction, was therefore purposefully selected to meet the study’s dual objectives of capturing future hydro-climatic variability and assessing flow seasonality and water resource implications in a highly sensitive alpine environment.

## Results and discussion

### Trend analysis

The results of the trend analysis for all observed meteorological station datasets (TMAX, TMIN, and PRCP) in the respective basins, using the nonparametric Mann–Kendall test and quantifying magnitude with Sen’s Slope. All trends were determined over the examination period, 1976–2005, with the level of statistical significance set at 90%. The results are listed below in Tables 7 and 8 for the observed stations.

**Table 7:** Mann–Kendall trend test for met. stations for the Chitral River Basin.

Chitral meteorological station			
**For maximum temperature (Tmax)**			
Time series	Z-score	Sen slope	Trend (90% Sig. level)
Jan	2.57	0.11	Increase
Feb	2.07	0.09	Increase
March	2.50	0.13	Increase
Dec	1.89	0.07	Increase
Annual	2.64	0.05	Increase
IX–XI	1.75	0.03	Increase
XII–II	3.10	0.10	Increase
**For minimum temperature (Tmin)**			
Time series	Z- score	Sen slope	Trend (90% Sig. level)
June	− 3.25	− 0.11	Decrease
July	− 2.43	− 0.07	Decrease
August	− 1.96	− 0.05	Decrease
Nov	− 2.18	− 0.04	Decrease
Annual	− 1.68	− 0.02	Decrease
VI–VIII	− 3.14	− 0.07	Decrease
**Precipitation (Prcp)**			
Time series	Z-score	Sen slope	Trend (90% Sig. level)
June	2.21	0.29	Increase
Sep	1.84	0.27	Increase
Annual	2.07	5.25	Increase
IX–XI	2.21	0.77	Increase

**Table 8:** Mann–Kendall trend test for met. stations for the Chitral River Basin.

Drosh meteorological station			
**For maximum temperature (Tmax)**			
Time series	Z-score	Sen slope	Trend (90% Sig. level)
Jan	2.00	0.08	Increase
March	1.86	0.08	Increase
XII–II	2.50	0.07	Increase
**For minimum temperature (Tmin)**			
Time series	Z-score	Sen slope	Trend (90% Sig. level)
March	2.25	0.08	Increase
XII–II	1.96	0.05	Increase
**Precipitation (Prcp)**			
Time series	Z-score	Sen slope	Trend (90% Sig. level)
April	1.74	2.12	Increase
Annual	1.93	6.27	Increase

The Mann–Kendall trend analysis for the Chitral River Basin (CRB) revealed a significant upward trend in both maximum temperature and precipitation across the annual scale, with parallel increases during autumn and winter. In contrast, a decreasing pattern was observed in minimum temperature, particularly during the monsoon season and over the overall annual time span from 1976 to 2005.

### Model calibration and validation

**Fig. 6:**
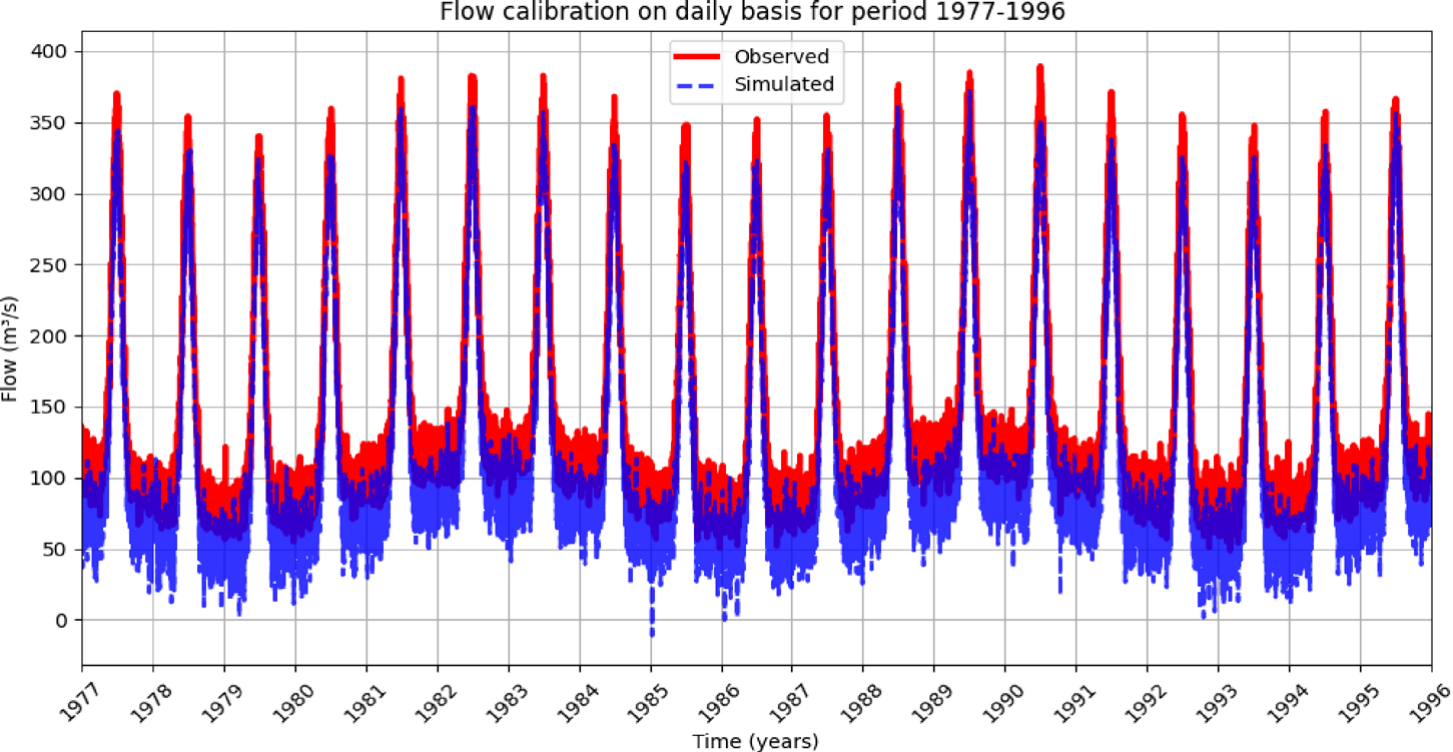
Observed and simulated daily discharge at the Chitral gauging station during the calibration period (1977–1996), in m³/s.

**Fig. 7:**
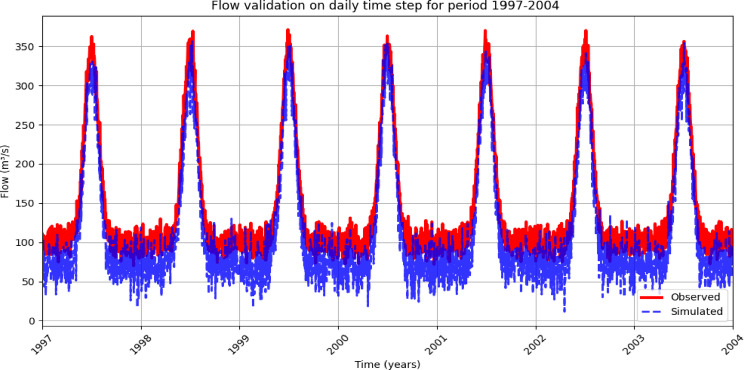
Observed and simulated daily discharge at the Chitral gauging station during the validation period (1997–2004), in m³/s.

The SWAT model performance for the Chitral River Basin was evaluated through calibration and validation using observed daily streamflow data. The calibration was conducted for the period 1977–1996, while an independent dataset for 1997–2004 was used for validation. Figures 6 and 7 illustrate the comparison between observed and simulated daily streamflow during the calibration and validation periods, respectively. The model successfully reproduces the seasonal flow dynamics and peak discharge magnitudes, particularly during the snowmelt-dominated high-flow months. Minor discrepancies are observed during low-flow periods, which are common in glacier-fed basins due to simplified groundwater and melt process representation.

**Fig. 8:**
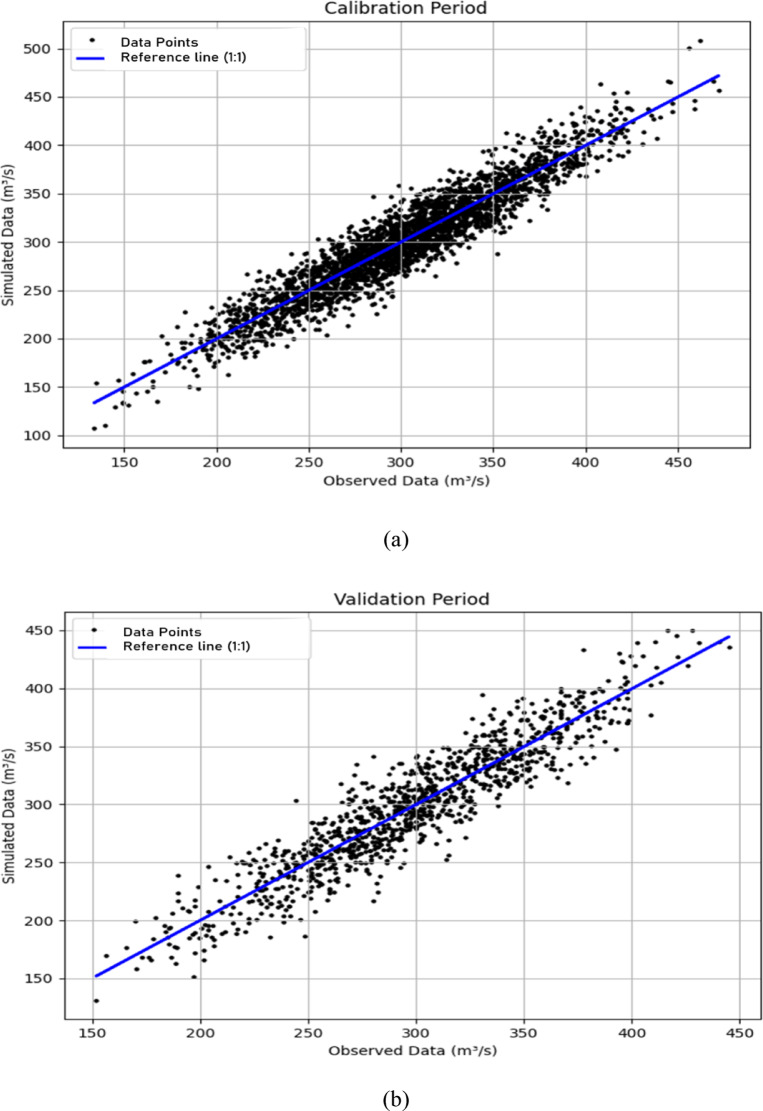
Scatter plots of observed versus simulated daily discharge at the Chitral gauging station for (**a**) calibration and (**b**) validation periods, in m³/s.

Figure 8 presents scatter plots of observed versus simulated streamflow for both calibration and validation periods, including a 1:1 reference line. The clustering of data points around the 1:1 line indicates good agreement between simulated and observed flows. Performance statistics demonstrate satisfactory model skill, with NSE values exceeding commonly accepted thresholds for daily streamflow simulation, confirming the model’s suitability for climate change impact assessment in the basin as shown in Table 9. Model calibration and validation were performed using the SUFI-2 algorithm within SWAT-CUP, with model uncertainty evaluated through the p-factor and r-factor metrics. These indicators quantify the degree to which observed streamflow is bracketed by the 95% prediction uncertainty and the average thickness of the uncertainty band, respectively. The obtained p-factor and r-factor values indicate satisfactory model performance for daily streamflow simulation in the Chitral River Basin, supporting the reliability of the model for climate change impact assessment.

**Table 9:** Model performance metrics.

Metric	Calibration (1977–1996)	Validation (1997–2004)
NSE	0.81	0.76
R²	0.84	0.79
PBIAS (%)	4.2	5.1
*p*-factor	0.78	0.72
r-factor	0.85	0.91

The apparent discrepancy between the mean annual flow (274 m³/s) and daily peak flows (8000–10,000 m³/s) is a direct consequence of the basin’s high‑altitude, snow/glacier‑melt regime. Daily peaks during May-August typically reach 8000–10,000 m³/s, while the annual mean remains lower due to very low flows in winter. The calibration was performed daily, and the performance metrics reported below reflect daily‑scale agreement.

Overall, the calibration and validation results indicate that the SWAT model adequately captures the hydrological behavior of the Chitral River Basin and provides a reliable basis for subsequent climate-driven streamflow projections.

### Projection in stream flows

**Fig. 9:**
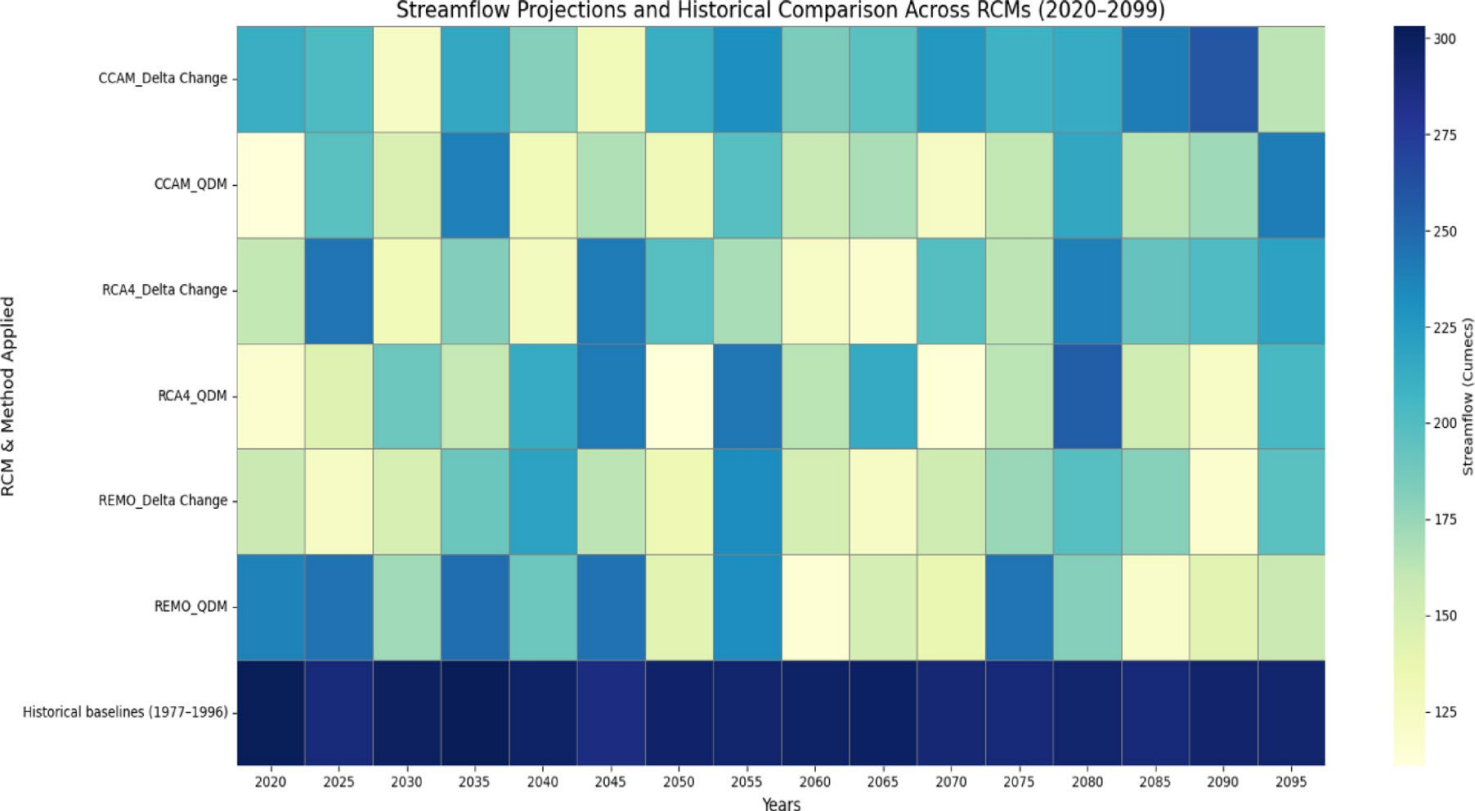
Comparison of projected streamflow trends across RCMs using heat map visualization.

**Fig. 10:**
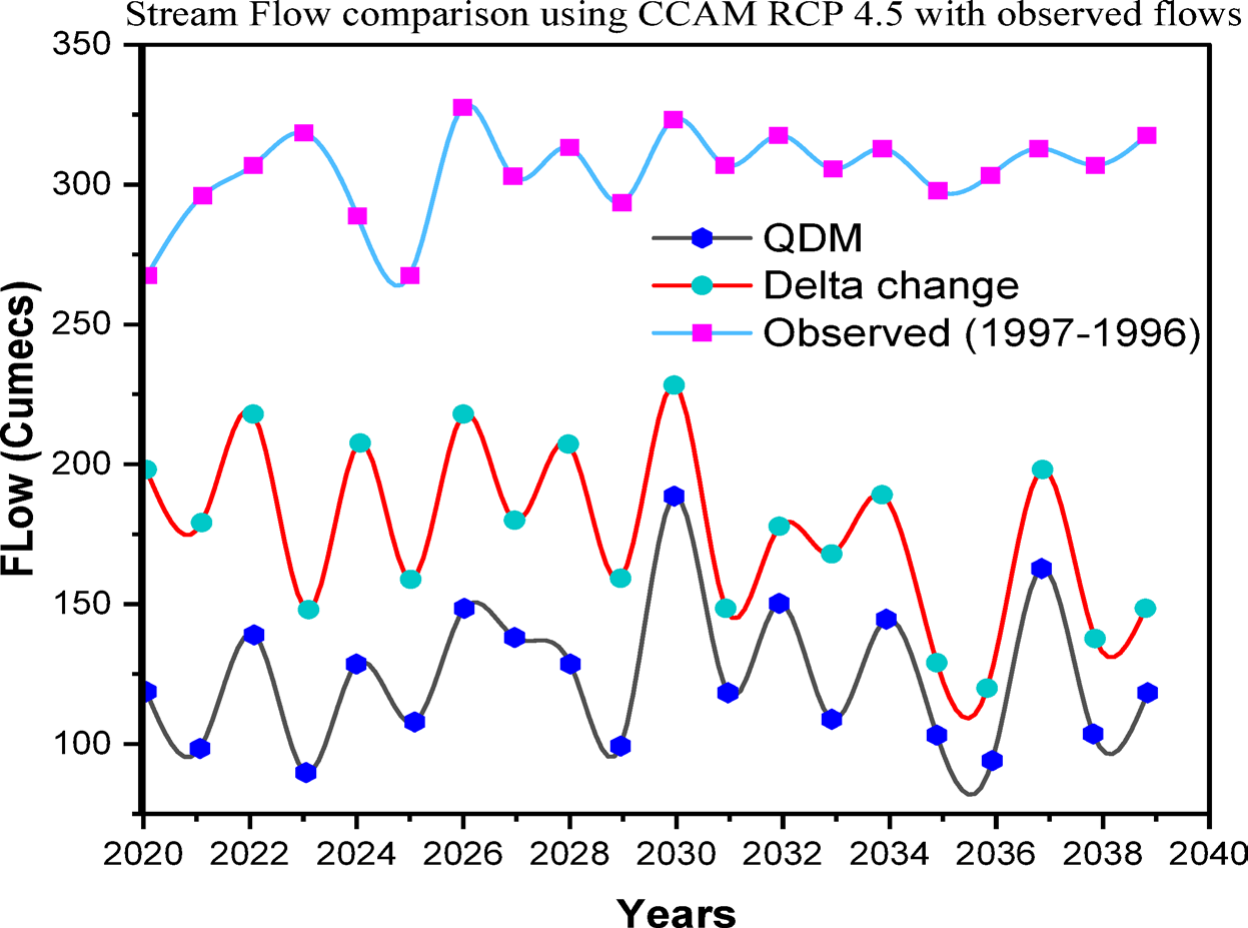
Streamflow projections RCP4.5.

**Fig. 11:**
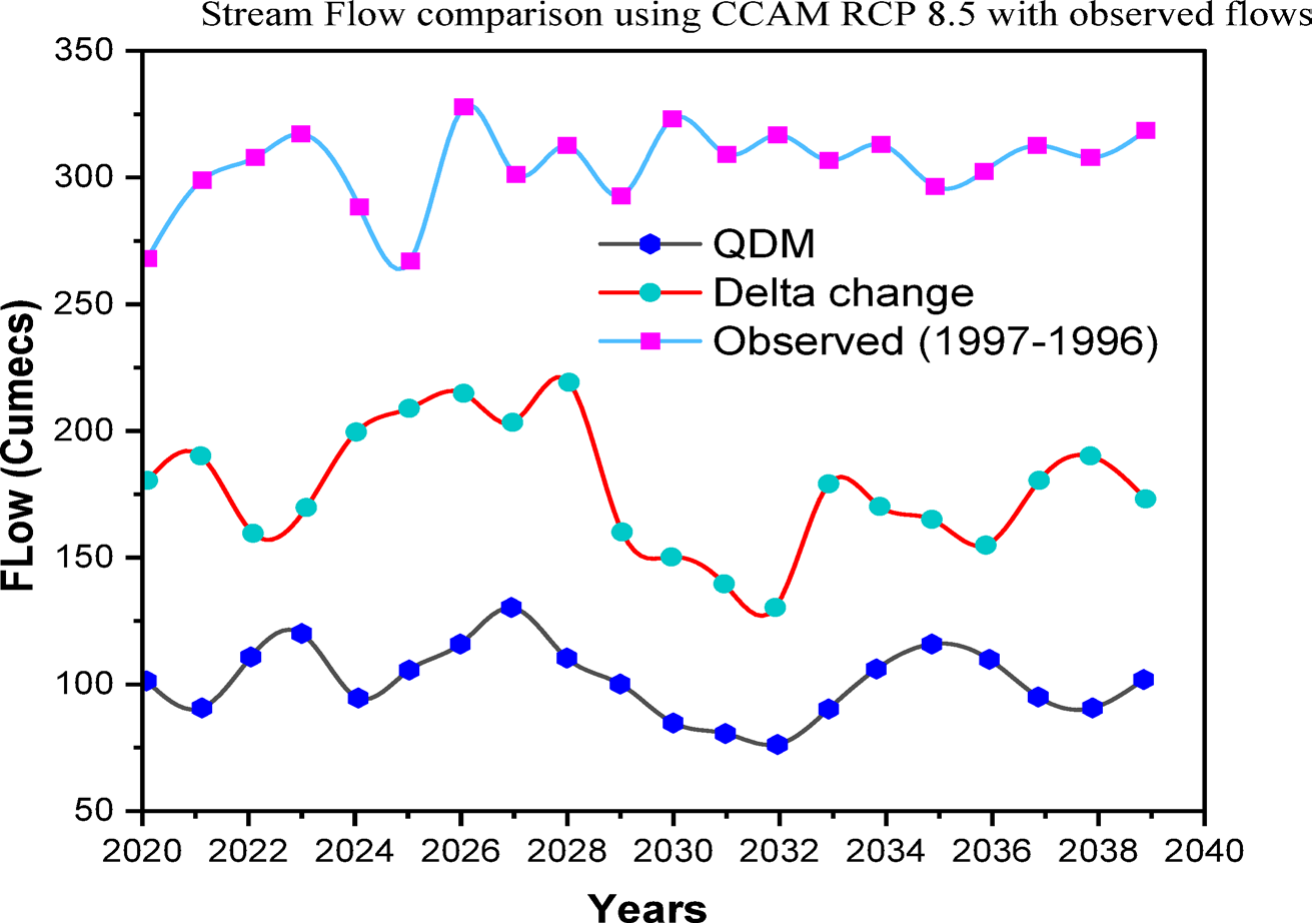
Streamflow projections RCP8.5.

**Fig. 12:**
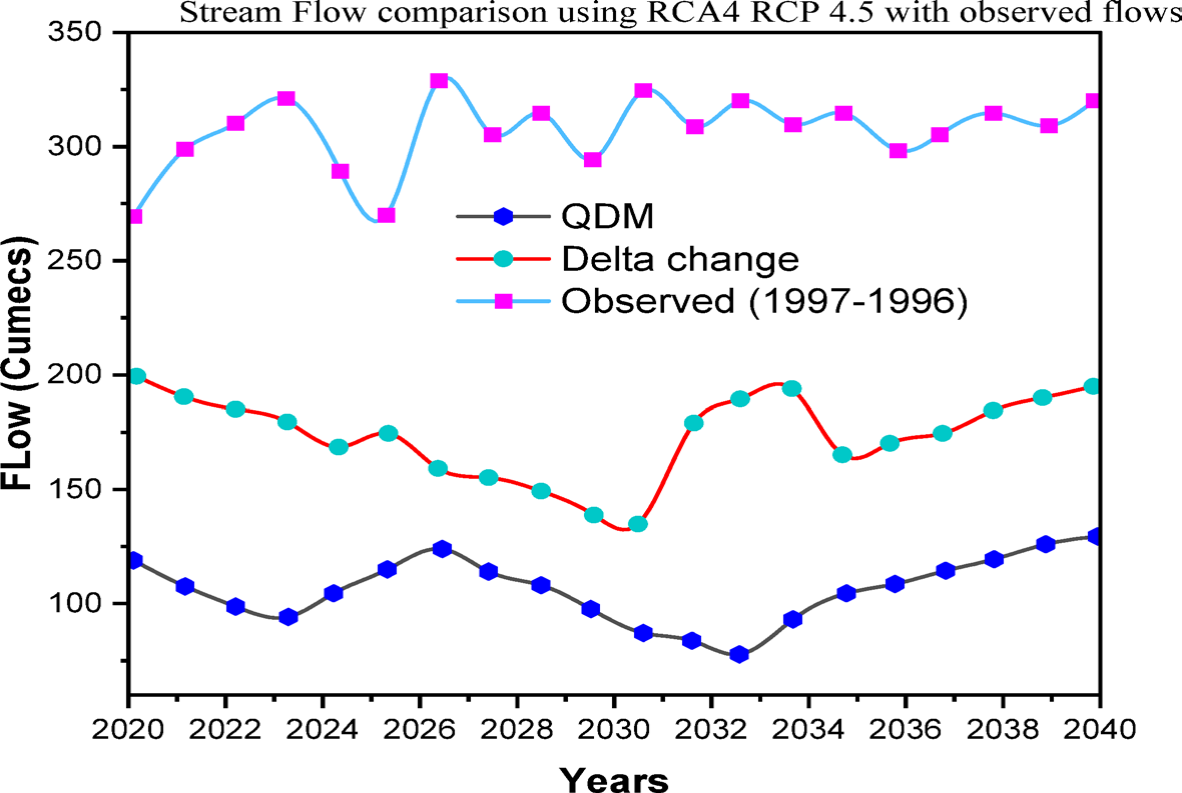
Streamflow projections RCP4.5.

**Fig. 13:**
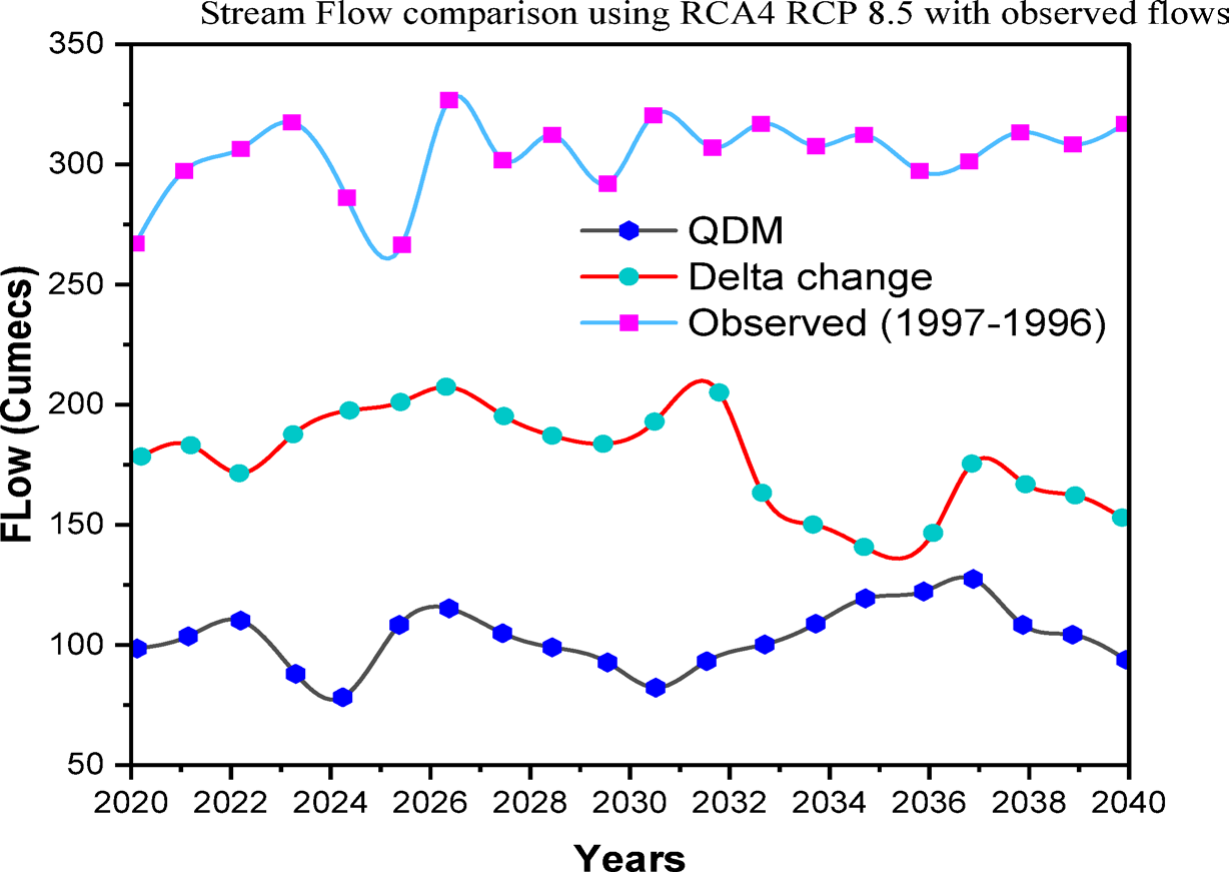
Streamflow projections RCP8.5.

**Fig. 14:**
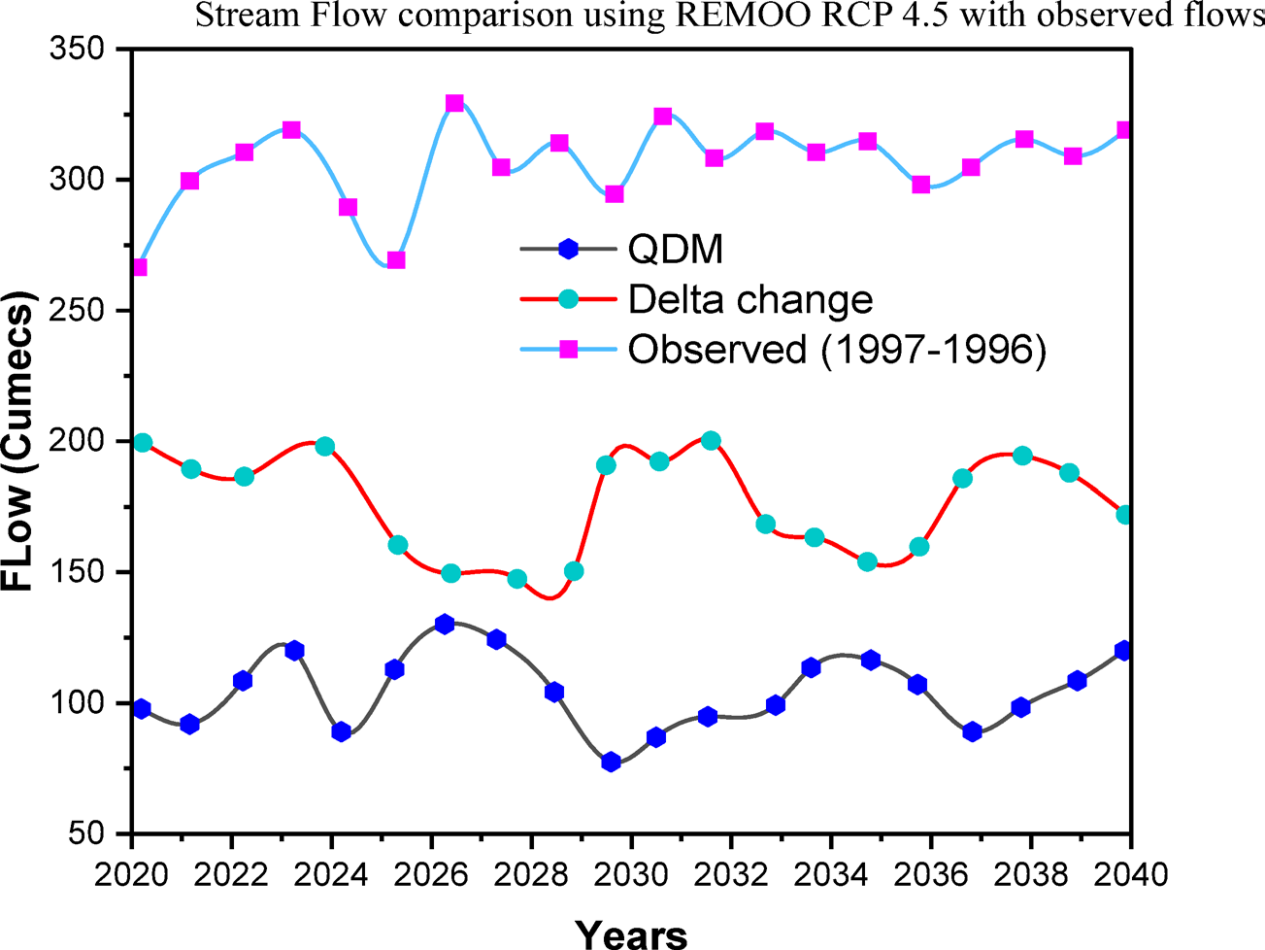
Streamflow projections RCP4.5.

**Fig. 15:**
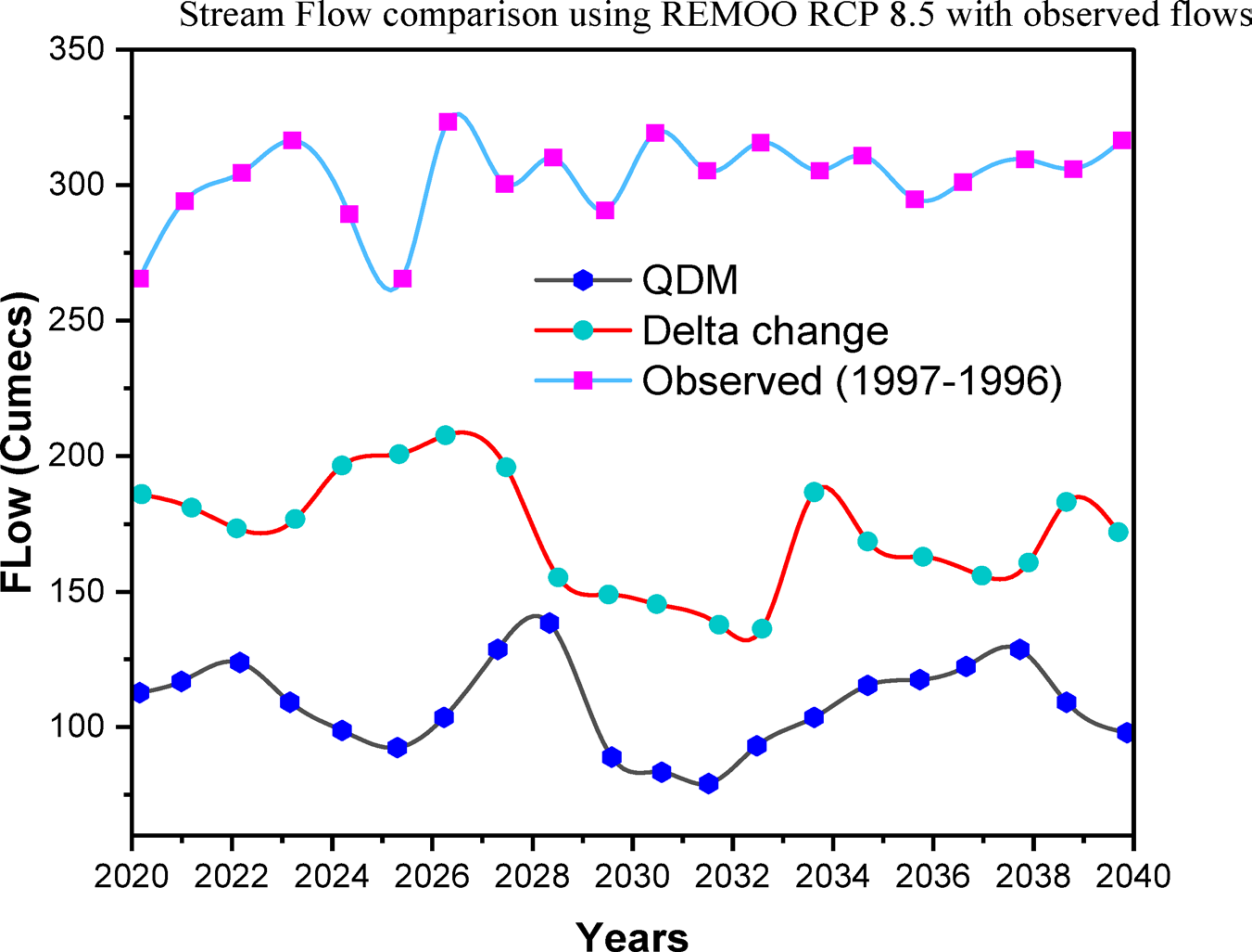
Streamflow projections RCP8.5.

The results are summarized in Table 10 and graphically shown in Figs. 9, 10, 11, 12, 13, 14, 15. Overall, the projections indicate substantial variability among RCMs, scenarios, and bias-correction methods. In several simulations, streamflow responses differ between early, mid-, and late-century periods, reflecting the combined influence of projected temperature and precipitation changes on basin hydrology. Because the SWAT configuration used here assumes static glacier area, these projected changes should be interpreted as climate-driven hydrological responses rather than direct evidence of progressive glacier depletion. Although some projected changes are directionally comparable to previous Upper Indus Basin studies that emphasize the importance of warming-induced snow and glacier melt, the present results should be interpreted cautiously because the adopted SWAT framework does not simulate dynamic glacier retreat or glacier mass balance. Therefore, the comparison with previous studies is limited to general hydro-climatic tendencies rather than explicit glacier-depletion mechanisms.

**Table 10:** Probable changes in flow in first, mid, and late century for the Chitral River Basin.

Difference in flows for different RCMs mean annual observed flow (1976–1996) = 274.3 m3 /s						
RCMs	2020–2039		2050–2069		2080–2099	
RCPs	(4.5)	(8.5)	(4.5)	(8.5)	(4.5)	(8.5)
CCAM	148.12–135.2 (− 8.72%)	125.09–112.25 (− 10.26%)	130.63–120.26 (− 7.93%)	127.96–121.83 (− 4.77%)	126.57–118.4 (− 6.46%)	121.8–117.92 (− 3.8%)
RCA4	106.73–98.81 (− 7.42%)	122.53–112.53 (− 8.16%)	129.5–114.18 (− 11.83%)	122.7–111.36 (− 9.24%)	128.51–114.26 (− 11.08%)	131.03–114.3 (− 12.76%)
REMOO 2009	177.2–143.92 (− 18.78%)	188–153.75 (− 18.21%)	178.41–44.96 (− 18.74%)	170.57–140.31 (− 17.74%)	167.4–135.29 (− 19.18%)	154.54–135.21 (− 12.50%)

The observed mean annual flow (274.3 m³/s) represents the long‑term average. Daily peaks during the snowmelt season (May–August) typically reach 8000–10,000 m³/s, as shown in Figs. 6 and 7. The model was calibrated using daily discharge data, and the performance metrics (“Projection in stream flows” section) reflect daily‑scale agreement.

To complement the quantitative findings presented in Table 10, a heat map visualization in Fig. 9 was developed to depict decadal streamflow projections from 2020 to 2099 across various RCMs and bias-correction methods. The figure highlights temporal variability and model disagreement, emphasizing fluctuations in projected discharge relative to the historical baseline (1977–1996). It can be observed that, under all scenarios, late-century streamflow is consistently lower than the baseline, indicating a significant drying trend across the Chitral River Basin. The REMO_QDM and RCA4. Delta change methods show relatively lower streamflow estimates throughout the century, whereas CCAM-based projections exhibit slightly less severe declines. The color pattern also reflects decadal variations, underscoring the importance of considering inter-decadal hydrological changes driven by climate forcing. This figure reinforces the declining trend observed in the tabular data and visually illustrates the divergence between models and correction methods.

The substantial spread across RCMs (ranging from − 3.8% to -19.18% by late century) reflects three sources of uncertainty: (1) different GCM boundary conditions driving each RCM, (2) varying representations of South Asian monsoon and westerly dynamics, and (3) interactions with bias correction methods. The REMO2009 model consistently projects the most severe reductions (18‑19% under both RCPs), while CCAM projects milder declines (4‑8%). This divergence is not systematically resolved by RCP scenario or time slice. Consequently, while the direction of change (declining late‑century flow) is robust across models, the magnitude remains highly uncertain. The increase‑then‑decrease pattern is not uniformly supported RCA4, for example, shows no clear early‑century increase in several simulations. This uncertainty fundamentally limits the strength of conclusions about peak flow timing.

#### Stream flow projection using CCAM RCM

The streamflow projections from the CCAM Regional Climate Model under the RCP4.5 and RCP8.5 scenarios show noticeable differences between the Delta change and Quantile Delta Mapping (QDM) methods. As shown in Figs. 10 and 11, the observed streamflow (1977–1996) consistently remains higher than the projected flows across both scenarios. The Delta change method yields relatively moderate reductions in streamflow. It remains closer to historical values, likely due to its simplified approach of uniformly adjusting historical data based on average climate-change signals. In contrast, the QDM method, which accounts for the statistical distribution of observed and modelled data, projects significantly lower flows. This disparity highlights the influence of bias correction techniques on future hydrological estimates. Under RCP8.5, which represents a more extreme emission scenario, both methods project higher flows compared to RCP4.5. However, QDM remains conservative throughout. These results emphasize the importance of selecting appropriate downscaling and correction techniques when modeling future hydrological conditions, as they can lead to markedly different outcomes and have substantial implications for water resource planning and climate adaptation strategies.

#### Stream flow projection using RCA4 RCM

Unlike the CCAM model, the RCA4 Regional Climate Model exhibits a more uniform pattern in streamflow reduction under both RCP4.5 and RCP8.5 scenarios for the period 2020–2039, as presented in Figs. 12 and 13. The projected flows derived from both the Delta change and QDM methods consistently fall below the historical observations, reflecting a notable decline in future water availability. Although the Delta change method yields slightly higher flow estimates than QDM, both methodologies show similar temporal trends, reinforcing the projection’s credibility. Under RCP8.5, a marginal increase in flow relative to RCP4.5 is observed, likely driven by intensified melting and increased runoff, yet these values remain substantially lower than baseline observations (1977–1996). The relatively narrow difference between the two correction methods in this model suggests less internal variability in RCA4 projections, which may imply more stable hydrological outcomes. These results emphasize the sensitivity of future streamflow’s to the choice of climate model and bias correction method and highlight the importance of accounting for these factors in regional water resource planning.

#### Stream flow projection using REMO 2009 RCM

In contrast to the other regional models, the REMO 2009 Regional Climate Model reveals a more pronounced deviation between projected and observed streamflow’s, as illustrated in Figs. 14 and 15. Both the Delta change and QDM methods produce streamflow estimates that are significantly lower than the historical baseline (1977–1996), with QDM projections appearing particularly conservative. The difference between Delta change and QDM outputs is more pronounced in REMO than in RCA4 and CCAM, suggesting greater sensitivity of this model to the applied bias-correction technique. Under both RCP4.5 and RCP8.5 scenarios, an apparent downward shift in flow values is observed, despite minor inter-annual variations. Notably, the flow reduction under RCP8.5 remains consistent with that under RCP4.5, indicating that even more extreme emissions may not offset the declining trend in streamflow due to underlying climatic and glacial dynamics. The substantial gap between modelled and observed values underscores the critical need for model calibration and highlights the REMO model’s conservative nature in projecting hydrological responses to climate change. These outcomes further reinforce the importance of applying multiple RCMs and bias correction strategies to capture the full spectrum of possible future hydrological behaviours in mountainous watersheds.

### Comparative analysis with similar studies in the upper Indus Basin

The projected climate changes in the Chitral River Basin (CRB) closely align with findings from several studies in the Upper Indus Basin, demonstrating similar trends in temperature and precipitation increases under RCP4.5 and RCP8.5 scenarios. In this study, our findings indicate a steady rise in seasonal temperatures, aligning well with earlier projections by Shah et al. (2020), who reported a temperature rise of 2.36–3.50 °C under RCP4.5 and 2.92–5.23 °C under RCP8.5^1^. This increase in temperature is also reflected in Anjum et al. (2019), who found a similar rise in the region, supporting the warming trends seen in our projections^63^. Furthermore, precipitation in the CRB is projected to increase during both the annual and seasonal timescales, aligning with results from Shah et al. (2020)^1^, who observed a yearly precipitation increment ranging from approximately 2.4–2.5% in RCP4.5 and up to 6.0% in RCP8.5 projections, and from Anjum et al. (2019)^64^, who noted similar increases in winter precipitation in the Upper Indus Basin. These temperature and precipitation trends are consistent with our findings in the CRB, confirming that climate change impacts are likely to affect both regions similarly.

In terms of streamflow projections, the CRB shows increases in mean annual flow of 7.42% to 12.76% for the first-, mid-, and late-century projections under RCP4.5 and RCP8.5. This increase in streamflow is primarily driven by rising temperatures and glacier melt in the region, a pattern similarly observed in previous studies. For instance, Shrestha et al. (2015)^65^ and Tahir et al. (2011)^66^ found that the Upper Indus Basin is projected to experience increased streamflow due to climate change, mainly driven by glacier melt and rising temperatures. Our study’s projections of streamflow increase for the first century are consistent with these findings, however, direct comparison is limited by differences in model structure (e.g., our static glacier area vs. dynamic glacier models used in Shrestha et al. 2015) and bias correction methods (QDM vs. simpler delta approaches in earlier studies) which emphasize the role of climate-induced changes in hydrological behavior in the region. This temporal pattern is consistent with a hypothesized transition from melt‑dominated to supply‑limited regimes, but direct simulation of glacier retreat would be required to confirm this interpretation.

### Practical implications of parametric calibration

Although climatic forcings play a significant role in shaping streamflow projections under RCP4.5 and RCP8.5 scenarios, calibrating key hydrological parameters within the SWAT model is also necessary to ensure their reliability. While a direct quantitative analysis of the impacts of individual parameters was not conducted in this study, the calibration process, which was based on SUFI-2 optimization and local sensitivity analysis, ensured that hydrologically significant parameters were carefully adjusted to reflect the hydrology of the Chitral River Basin, which is characterized by considerable snow and glacier coverage.

Prior modeling research has shown that early-summer peak flows are influenced by parameters such as SMFMX (maximum snowmelt rate), which are expected to increase in warmer climates. As such, the baseflow recession constant, or ALPHA_BF, regulates the persistence of flow during dry spells, thereby affecting groundwater-sustained discharge. During high precipitation events, parameters such as CN2 (curve number) and SOL_K (saturated hydraulic conductivity) govern runoff response and infiltration dynamics, thereby affecting soil moisture retention and flood potential as shown in Table 11 which lists the calibrated parameters and their physical relevance.

Although long-term streamflow responses to climate scenarios were the focus of this work, the model’s ability to reasonably replicate actual hydrographs and flow seasonality was aided by the choice and calibration of these parameters. This underscores the importance of parameter sensitivity in determining future estimates. Furthermore, these calibrated parameters help ensure that modelled responses, such as seasonal changes or greater variability, reflect actual system behavior rather than being artifacts of parameter misrepresentation. A more focused parametric evaluation of influence in subsequent research could help confirm the usefulness of climate-hydrology models in glacier-fed areas.

**Table 11:** Calibrated SWAT parameters for the Chitral River Basin.

Parameter	Description	Calibrated Range
CN2	SCS runoff curve number	−0.15 to + 0.10
ALPHA_BF	Base flow recession constant	0.02–0.25
SOL_K	Saturated hydraulic conductivity (mm h^−1^)	5–40
SMFMX	Maximum snowmelt factor (mm °C^−1^ day^−1^)	3.5–6.0
SMFMN	Minimum snowmelt factor (mm °C^−1^ day^−1^)	1.5–3.0
ESCO	Soil evaporation compensation factor	0.6–0.9
GW_DELAY	Groundwater delay time (days)	30–120

## Conclusion and discussion

### Conclusion

This study evaluated the impacts of projected climate change on streamflow dynamics in the Chitral River Basin using a multi-RCM, bias-corrected SWAT modeling framework. By isolating climate-driven effects under naturalized flow conditions, the analysis provides insight into both transient and long-term hydrological responses of a glacier-fed, high-mountain river system. The principal findings and implications of the study are summarized as follows:

- The Chitral River Basin is governed by a temperature-dependent glacier–snowmelt-dominated hydrological regime, making streamflow highly sensitive to climatic warming.
- Climate projections indicate mid-century increases in mean annual streamflow of up to ~ 20% under both RCP4.5 and RCP8.5, primarily driven by accelerated snow and glacier melt.
- Late‑century streamflow declines are projected under several simulations, but these reflect climate‑hydrological responses under static glacier area. The magnitude of additional reductions due to progressive glacier retreat remains unquantified in this study.
- Seasonal peak flows shift earlier toward June-July, altering runoff timing and increasing the likelihood of summer water scarcity in downstream regions.
- The projected changes represent climate-driven impacts under naturalized flow conditions like peak discharge characteristics.

A key limitation is the large spread across RCM projections (late‑century changes ranging from − 3.8 to − 19.2%), which precludes precise quantification of future flow magnitudes despite robust directional signals for seasonal timing shifts. Overall, the results highlight the transient nature of meltwater-driven flow gains and underscore the need for adaptive, climate-resilient water resource planning in high-mountain river basins.

### Limitations of RCMs and bias correction techniques

While the RCMs provide a higher spatial resolution for climate projections, they are inherently dependent on the Global Climate Models (GCMs) used for boundary conditions. These GCMs themselves carry a degree of uncertainty that propagates through the RCM outputs, leading to biases. These biases can lead to discrepancies when comparing simulated data with observed data. Additionally, RCMs typically have a resolution of 50–100 km. Although they offer a more localized representation of climate, this resolution is often insufficient to capture small-scale variations, particularly in regions with complex terrain, such as the Chitral River Basin. Localized topographical features, such as mountain ranges, can significantly influence precipitation and temperature patterns, which might not be fully represented in RCM projections. Another key limitation lies in the bias correction methods. In this study, we used the CMhyd bias-correction framework, which assumes that past biases in RCM or GCM outputs will persist in the future. This assumption may not always hold, particularly when future climate dynamics diverge from past trends. Moreover, the process of bias correction is typically focused on annual averages and seasonal trends. However, short-term variability and extreme weather events are often underrepresented in bias-correction methods, potentially limiting their application to flood forecasting or drought prediction. Finally, despite efforts to address data gaps, the issue of missing data or incomplete records remains a challenge in both RCM outputs and observational datasets. Gaps in temperature or precipitation records can influence the accuracy of both the raw RCM simulations and the final bias-corrected outputs^67^. Uncertainty also arises from SWAT model structure, parameter equifinality, and the representation of snow and glacier melt using a temperature-index approach. Climate forcing uncertainty linked to RCM selection further contributes to projection spread. Additionally, the absence of dynamic glacier mass balance modeling limits the model’s ability to explicitly represent future glacier retreat and its hydrological consequences, which increases uncertainty in long-term streamflow projections.

## Data Availability

Hydro-meteorological datasets used in this study were obtained from the Pakistan Meteorological Department (PMD) and the Water and Power Development Authority (WAPDA). Due to institutional restrictions, these data are not publicly available but can be provided by the corresponding author upon reasonable request. Regional Climate Model (RCM) datasets were retrieved from the Earth System Grid Federation (ESGF) and are publicly accessible via: https://esgf-node.llnl.gov. Processed SWAT model output data are available from the corresponding author upon request.
